# Propensity for Early Metastatic Spread in Breast Cancer: Role of Tumor Vascularization Features and Tumor Immune Infiltrate

**DOI:** 10.3390/cancers13235917

**Published:** 2021-11-25

**Authors:** Mario Rosario D’Andrea, Vittore Cereda, Luigi Coppola, Guido Giordano, Andrea Remo, Elena De Santis

**Affiliations:** 1 Clinical Oncology Unit, San Paolo Hospital, Largo Donatori del Sangue 1, Civitavecchia, 00053 Rome, Italy; mariorosario.dandrea@aslroma4.it; 2 Unit of Anatomy, Pathological Histology and Diagnostic Cytology, Department of Diagnostic and Pharma-Ceutical Services, Sandro Pertini Hospital, 00157 Rome, Italy; luigi.coppola@aslroma2.it; 3 Unit of Medical Oncology and Biomolecular Therapy, Department of Medical and Surgical Sciences, University of Foggia, Policlinico Riuniti, 71122 Foggia, Italy; guido.giordano@unifg.it; 4 Pathology Unit, Mater Salutis Hospital, ULSS9, Legnago, 37045 Verona, Italy; andrea.remo@aulss9.veneto.it; 5 Department of Anatomical, Histological, Forensic Medicine and Orthopedic Sciences, Sapienza University of Rome, 00185 Rome, Italy; elena.desantis@uniroma1.it

**Keywords:** tumor angiogenesis, breast cancer, cytokines, tumor microenvironment, metastasis

## Abstract

**Simple Summary:**

Neoangiogenesis and different components of tumor microenvironment exert crucial roles in early metastatic spreading of breast cancer. Due to the paucity of available factors for breast cancer that can estimate the risk of metastasis or response to a given treatment, there is an important need for new, additional biomarkers of the tumor stroma that can be prognostic and predictive at the early stages of the disease. This review is focused on the current understanding of breast tumor environment characteristics (tumor vasculature and immune infiltrate) in order to incorporate them in diagnostic algorithms together with the assessment of hormone receptors (ER and PR) and of HER2 status.

**Abstract:**

Breast cancer is a complex and highly heterogeneous disease consisting of various subtypes. It is classified into human epidermal growth receptor 2 (HER-2)-enriched, luminal A, luminal B and basal-like/triple negative (TNBC) breast cancer, based on histological and molecular features. At present, clinical decision-making in breast cancer is focused only on the assessment of tumor cells; nevertheless, it has been recognized that the tumor microenvironment (TME) plays a critical biologic role in breast cancer. This is constituted by a large group of immune and non-immune cells, but also by non-cellular components, such as several cytokines. TME is deeply involved in angiogenesis, immune-evasion strategies, and propensity for early metastatic spread, impacting on prognosis and prediction of response to specific treatments. In this review, we focused our attention on the early morphological changes of tumor microenvironment (tumor vasculature features, presence of immune and non-immune cells infiltrating the stroma, levels of cytokines) during breast cancer development. At the same time, we correlate these characteristics with early metastatic propensity (defined as synchronous metastasis or early recurrence) with particular attention to breast cancer subtypes.

## 1. Introduction

**Figure 1:**
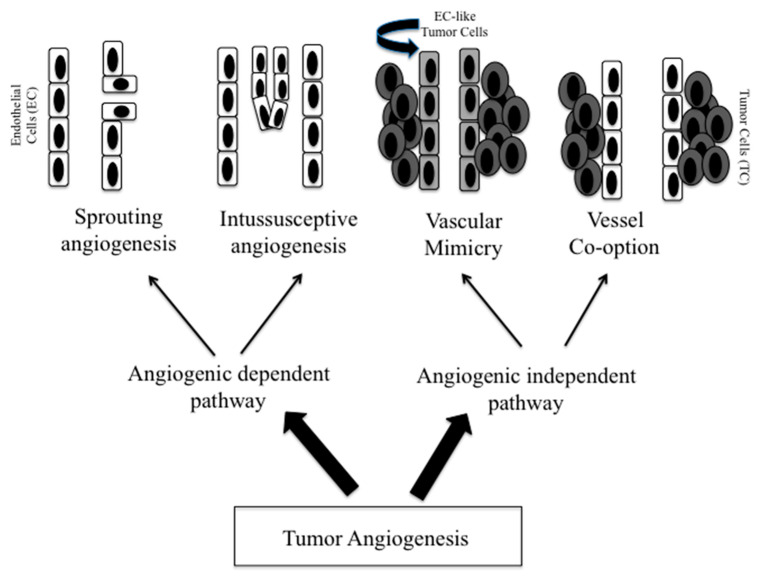
Different forms of tumor neovascularization. The figure shows the angiogenic-dependent and -independent pathways, through which tumors may provide their blood supply. The two angiogenic-dependent pathways are: (1) sprouting angiogenesis: a dynamic paracrine crosstalk between tumor cells and tumor microenvironment that generates a vascular sprout arising from a pre-existing vessel; (2) intussusceptive angiogenesis: an alternative angiogenic process that consists of the invagination of the capillary walls into the vascular lumen of a pre-existing vessel. The two angiogenic-independent pathways are: (1) vascular mimicry: some tumor cells may generate channels that mimic the function of vessels. These tumor cells have a high plasticity that allows them to acquire endothelial cell-like traits; (2) vessel co-option: tumor cells, migrating along the preexisting vessels of the host organ, hijack the surrounding normal vessels with preservation of the organ pre-existing architecture.

Breast cancer (BC) is the second most frequently diagnosed malignancy and the second leading cause of cancer-related death among women worldwide [1]. Although the incidence of distant relapse has been shown to be decreasing and overall survival for patients with recurrent disease have improved, 20–30% of patients with early breast cancer still die of metastatic disease [2]. The process of metastasis comprises of a series of sequential steps, requiring the development of an invasive phenotype through a progressive loss of the epithelial architecture: tumor cells need to escape from immune surveillance, invade lymphatic or blood vessels, and remotely prepare the microenvironment at the future metastatic site [3,4]. Large cohort patient studies demonstrated that tumor cells dissemination could already occur at the early stages of tumor development, such as the pre-invasive stage “ductal carcinoma in situ” (DCIS) and proceed in parallel to or independently from tumor invasion into the surrounding stroma [5]. Indeed, circulating tumor cells may be found in the peripheral blood or in the bone marrow of approximately 13–25% of patients with both pre-invasive DCIS and invasive ductal carcinomas [6]. Interestingly, among patients with pure DCIS, although just 5–30% experience an ipsilateral recurrence and about half of these recurrences are invasive cancer, the 5-year risk of death after treatment ranges from 1.3% to 2.7%, suggesting that prevention of in-breast invasive recurrences does not prevent death from breast cancer [7,8,9]. It has been established that the constitution of a supporting vasculature network is a crucial step for tumor progression from a benign to a malignant state [10], therefore representing the prerequisite for tumor growth and dissemination [11,12]. Many observations suggest that angiogenesis (sprouting and intussusceptive), the development of new blood vessels following the proliferation of the endothelial cells of pre-existing vessels, may initiate very early since the start of ductal hyperplasia [13,14] and the pattern of extent of vascularization around DCIS (diffuse stromal vascularity versus vascular rim around the ducts) may determine the transformation from in situ to invasive carcinoma [15]. There is however evidence that both primary tumors and metastases are able to progress without angiogenesis. As already reported, two alternative tumor blood supplies are vasculogenic mimicry and vessel co-option [16]. Figure 1 depicts the different forms of tumor neovascularization.

Of note, it has been recently demonstrated that tumor cells may reach the bloodstream through specific micro-anatomical structures called tumor microenvironment of metastasis (TMEM), a three-cell complex composed of a perivascular macrophage, a tumor cell expressing high levels of actin-regulatory protein mammalian enabled (Mena), and an endothelial cell [17,18]. Indeed, it has been showed that, depending on breast cancer specific sub-type, stromal components, such as tumor-associated macrophages (TAMs) and/or activated fibroblast (Cancer Associated Fibroblast—CAF) are involved alone or in combination with immune cells to trigger the transition from pre-invasive DCIS to invasive carcinomas and create a complex system that supports tumor dissemination [19]. A previous report showed that tumor cells bring from the primary site their own soil (stromal cells), as a provisional stroma, to the second site, in order to predispose their migration [20]. Furthermore, at the same time, tumor cells not only drive the recruitment of stromal cells and immune cells from the surrounding tissues, but also affect the systemic environment, particularly the bone marrow through haematopoiesis alterations [21]. Once circulating monocytes have been recruited to the tumor stroma, they differentiate in macrophages and move together with tumor cells toward blood vessels, attracted by perivascular fibroblasts. At the blood vessels site, macrophages finally differentiate into perivascular macrophages, regulating vascular leakiness and cancer cell intravasation [22].

BC is a heterogeneous disease composed of different subtypes with distinctive histological and molecular characteristics. Nowadays, clinical decision-making is focused on the assessment of hormone receptors (estrogen (ER) and progesterone (PR) receptors) and of the epidermal growth factor receptor (HER-2) status, by a combination of immunohistochemical and in situ hybridization techniques on tumor cells. Nowadays, it is recognized that BC is a complex dynamic ecosystem and its development and progression do not only depend on tumor cell genetic and epigenetic aberrations but are also controlled by the tumor microenvironment (TME) through continuous mutually dynamic interactions. Along tumor development and invasion, the adjacent stroma is subjected to profound alterations in composition (cellular and non-cellular components) and is often involved in immune-evasion strategies and propensity for early metastatic spread, impacting on prognosis and prediction to treatment response [4,23]. In this review, starting from a morphological point of view assessed by immunohistochemistry, we focused our interest on the correlations between some tumor environment characteristics (tumor vasculature features and immune infiltrate) and early metastatic propensity (defined as synchronous metastasis or early recurrence) with particular attention to BC subtypes.

## 2. Tumor Vasculature

### 2.1. Sprouting and Intussusceptive Angiogenesis

#### 2.1.1. Molecular Mechanisms of Sprouting and Intussusceptive Angiogenesis

The availability of an adequate tumor vasculature is crucial for tumors in order to grow, maintain and progress over time and it is regarded as a hallmark in cancer development [3]. Vascular homeostasis is regulated by a large number of pro- and anti-angiogenic factors. When these are in balance, the vasculature is quiescent and endothelial cells are non-proliferative. The “angiogenic switch” is a process characterized by the alteration of this balance, when pro-angiogenic signaling is dominating. It can be triggered either by the additional genetic alterations of tumor cells, leading to increased proliferation, hypoxia, and over-expression of pro-angiogenic factors, or by tumor-associated inflammation and recruitment of immune cells [24]. The switch depends on increased production of one or more of the positive regulators of angiogenesis, such as vascular endothelial growth factor (VEGF), acid and basic fibroblast growth factor (βFGF and βFGF), transforming growth factor-β1 (TGFβ1), angiopoietins, hypoxia-inducible factors (HIF-1 and HIF-2), and mast cell-derived metalloproteinases. Among the others, VEGF is one of the most potent inducers of angiogenesis. It is produced and secreted by tumor cells and stroma cells; under hypoxic conditions, it activates endothelial cell proliferation and migration (through the overexpression of MMP-2 and MMP-9) and induces vascular permeability that boosts hypoxia, which enhances recruitment of stromal cells, endothelial cells, immune and inflammatory cells, from the surrounding tissues [25]. βFGF is released by tumor and stroma cells or is mobilized from extracellular matrix (ECM) and exerts its effects on endothelial cells via a paracrine signaling, by inducing their proliferation and the secretion of MMPs and collagenase [26]. The different roles of PDGFβ, TGFβ and angiopoietins are differently discussed below. It has also been demonstrated that one of the main stress factors that activates neo-angiogenesis during tumor development is hypoxia. Increased oxygen consumption, due to hyper-proliferation of tumor cells, leads to enhanced transcriptional activity of HIFs, which ultimately promotes the upregulation of several pro-angiogenic genes, especially in the endothelium [27].

**Figure 2:**
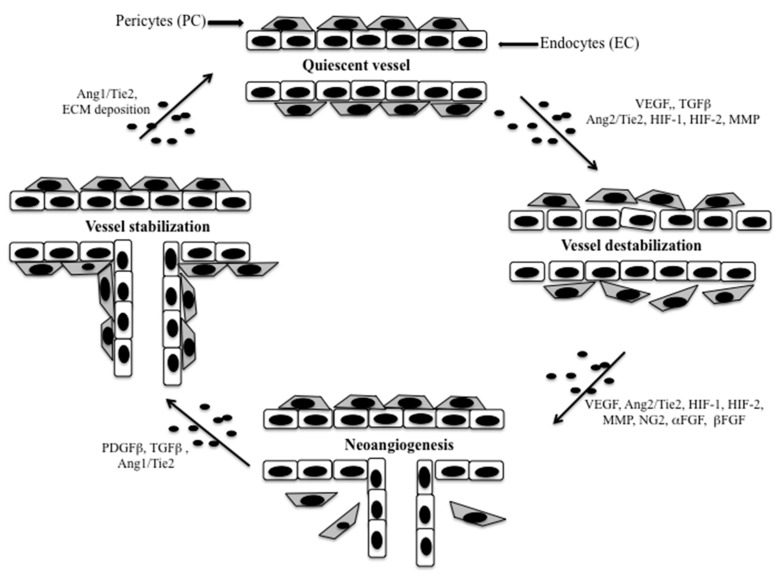
Crosstalk between endothelial cells and pericytes during sprouting angiogenesis: The formation of newly endothelial tubules from pre-existent vessels begins with aberrations in endocytes (ECs)-pericytes (PCs) signaling networks, which induce PCs-ECs dissociation and degradation of basement membrane by mean of proteolytic activities to allow cell migration and for creating space in the matrix. New vessel maturation is characterized by pericyte recruitment, functional pericyte investment of the endothelium, and assembly of ECM components. In this context, PC release pro-angiogenic factors, such as vascular endothelial growth factor (VEGF), acid and basic fibroblast growth factor (βFGF and βFGF), transforming growth factor-β1 (TGFβ1), hypoxia-inducible factors (HIF-1 and HIF-2) and mast cell-derived metalloproteinases (MMP), leading to degradation of the basement membrane, vasodilatation, increased vessel permeability and endothelial tube formation. The recruitment of pericytes, surrounding immature sprouting vessels with consequently pericyte coverage, is mediated principally by the release of TGFβ and platelet-derived growth factor β (PDGFβ) by ECs, which activates the corresponding receptor on pericytes. Conversely to ECs, tumor cell derived PDGFβ may induce the promotion of the pericyte to fibroblast activated cells (CAF) transition, inducing tumor invasion and metastasis through paracrine TGFβ signaling and mechanical pressure. It has also been showed that the angiopoietin (Ang1 and Ang2) signaling pathway may be crucial in the PCs-ECs crosstalk. Ang1 is secreted by periendothelial cells (pericytes, fibroblasts, smooth muscle cells, macrophages) and induces vessel stabilization and pericyte coverage, while Ang2 is principally secreted by activated ECs and promotes pericytes detachment inducing ECs, sprouting and tumor cell intravasation.

It has been demonstrated that in BC a high microvascular density has been largely associated with adverse clinical-pathological features (large tumor size, high histological grade, lymph node metastasis and poor prognosis) [28]. Overall, in BC, tumor vasculature derives from angiogenesis, and newly formed vessels are generated from a series of temporally distinct events that mostly involve endothelial cells (EC) and pericytes [29] (Figure 2).

From this point of view, we focus our attention on the interplay between endothelial cells and pericytes, while the contribution of other stroma cells in tumor angiogenesis is discussed in the Section 3.1.2. Indeed, a large amount of data support a central role of multiple tumor microenvironment components, particularly pericytes, by releasing pro-angiogenic factors [30,31]. The formation of newly endothelial tubules from pre-existent vessels begins with aberrations in EC-pericytes signaling networks, which induce pericyte-EC dissociation and degradation of basement membrane by means of proteolytic activities to allow cell migration and for creating space in the matrix. This process is regulated directly and indirectly by a variety of pro- and anti-angiogenic signaling, including VEGF and angiopoietin pathways (Ang1-2) [32,33]. The recruitment of pericytes surrounding immature sprouting vessels with consequently pericyte coverage is mediated principally by the release of TGFβ and platelet-derived growth factor β (PDGFβ) by ECs, which activates the corresponding receptor on pericytes [34]. Conversely to ECs, tumor cell-derived PDGFβ may induce the promotion of the pericyte to fibroblast activated cells (CAF) transition, a process similar to what happens in the epithelial to mesenchymal transition (EMT). These stromal activated cells are known to induce tumor invasion and metastasis through paracrine TGFβ signaling and mechanical pressure [35,36,37]. It has also been showed that the angiopoietin (Ang1 and Ang2) signaling pathway may be crucial in the pericyte-ECs crosstalk. Ang1 is secreted by periendothelial cells (pericytes, fibroblasts, smooth muscle cells, macrophages) and induces vessels stabilization and pericyte coverage, while Ang2 is principally secreted by activated ECs and promotes pericytes detachment inducing ECs sprouting and tumor cells intravasation [38]. The loss of pericytes, reducing vessel coverage and stability, induces an impairment of the tumor blood flow that is associated with the emergence of tumor hypoxia and increased expression of HIF-1α [39]. This process may alter the intrinsic characteristics of tumor cells increasing their aggressiveness and metastasis propensity [40]. Therefore, EC continuous stimulation, as demonstrated in tumor models expressing constitutive activating Tie2 (an angiopoietin receptor) mutations, induces the formation of dilated ectatic vessels instead of regular structures with disrupted apico-basal polarity and altered interactions with pericytes [41]. However, Tie2 expression has been observed even in macrophages, vascular smooth muscle cells and pericytes [42] and recently it has been suggested that Tie2-expressing pericytes may have a role in limiting endothelial sprouting, while their depletion results in enhancing tumor growth. These data depict a new vision of the Ang/Tie2 EC-pericyte crosstalk indicating it as a bidirectional relationship [43]. Pericytes and vascular smooth muscle cells belong to the mesenchymal cell lineage, as well as fibroblasts and macrophages and their heterogeneity may be derived from their capability to differentiate into each other throughout the neo-angiogenesis process. At the same time, the imbalanced expression or activation of several signaling components (Ang1/Ang2-Tie2, VEGF-VEGFR2, PDGFβ-PDGFRβ) would induce a higher or lower excess of immature sprouting capillaries. As a result of this dynamic paracrine crosstalk between tumor cells and TME, a vascular sprout arises from a pre-existing vessel; this process, also defined as “sprouting angiogenesis” (SA), was for long time thought to be the only angiogenesis way for vascular growth in tumors. An alternative angiogenic way to increase the tumor blood supply has been described in murine models and consists in the invagination of the capillary walls into the vascular lumen. This process of splitting of vessels, called “intussusceptive angiogenesis” (IA), is achieved at lower energy costs as compared to vessel sprouting [44]. IA, unlike SA, is a faster process that does not promote basement membrane disruption and becomes predominant in larger tumors. Interestingly, it has been suggested that αSMA positive cells (one of pericyte/periendothelial cell markers—the α-smooth muscle actin) have a critical role in intussusception as part of the angio-adaptive mechanism, and that the upregulation of αSMA could be due to PDGFβ and its receptors overexpression [45].

#### 2.1.2. Immunohistochemical Assessment of Sprouting and Intussusceptive Angiogenesis

All these changes in tumor vasculature can be evaluated by histological assays using ECs and pericytes markers but, as IA is an intravascular process, its specific assessment is very challenging. To date, only a few descriptive methods (scanning electron microscopy with vascular corrosion casts, light microscopy, confocal laser scanning microscopy) have been performed to recognize IA (44). Consequently, there are no data reported in the literature regarding the relationship between the presence and extension of IA in tumors and its impact on clinical outcomes. Therefore, the majority of information on the impact of angiogenesis on the propensity of early metastatic spread and clinical outcome in BC derives from SA and microvascular density (MVD) counting protocols, which are the morphological gold standard to assess the neovasculature in human tumors. This method requires the use of specific markers of vascular endothelium (traditionally, pan-endothelial markers as CD31, CD34 and von Willebrand factor/factor VIII antigen) and of immunohistochemical procedures to visualize microvessels. High MVD expression levels have been related to poor prognosis in early BC, as highlighted in a meta-analysis of earlier studies [46]. Indeed, high MVD significantly predicted poor survival [Risk Ratio = 1.99 for Relapse Free Survival (95% CI, 1.33-2.98) and 1.54 for Overall Survival (95% CI, 1.01-2.33)]. Between-study variations could result from patient selection criteria, techniques to stain and count microvessels, and cutoff selection. MVD is a significant although weak prognostic factor in women with breast cancer [46]. Because the employed markers are specific for all endothelia and do not distinguish new tumor-induced vessels from pre-existing vessels, many other markers and methods to quantify tumor vascularity in histopathological sections have been proposed over the time (i.e., proliferating MVD, vascular proliferation index VPI, and microvessel pericyte coverage index MPI), without any improvement [47]. Therefore, standardization of MVD assessment is needed. Recently, many attempts to correlate specific BC molecular subtypes with tumor angiogenesis (as measured with MVD, proliferating MVD, and VPI) and its impact on prognosis have been also performed, but the results were still inconclusive [48,49,50,51]. Thus, it can be thought that there should not be clear differences in tumor vascularity between subtypes, but level of vascularization could help to discriminate more aggressive tumors inside each subtype. As reported in a brilliant comprehensive study [51], a clear difference in MVD between basal-like and Luminal A primary tumors was not found, while high MVD was associated with poorer survival for patients with the Luminal A subtype (*p* = 0.038), but not for patients with the basal-like subtype (*p* = 0.7). Furthermore, a higher number of proliferating vessels were found among basal-like tumors compared to Luminal A tumors, although neither proliferating MVD nor VPI was associated with BC specific survival. Table 1 shows the results of this study.

To date, there have been no strictly pericyte-specific markers to assess the degree of vascular coverage and all markers currently used are dynamic in their expression in conjunction with the evolving pericyte-EC crosstalk. Thus, over time, different molecules have been evaluated individually or in combination, including PDGFRβ, Desmin, chondroitin sulfate proteoglycan 4 (NG2), and αSMA, and it has been shown that level of expression and type of marker exhibited by pericytes correlates with the degree of pericyte differentiation and coverage (generally measured as microvessel pericyte coverage index—MPI) [28]. However, some experimental data suggest that perivascular PDGFRβ+ cells are progenitors, partly recruited from the bone marrow, having the ability to differentiate into mature desmin+, NG2+ and aSMA+ pericytes/vascular smooth muscle cells [32]. NG2 proteoglycan (also called CSPG4) is supposed to play a relevant role in pericyte proliferation and motility, as well as in pericyte-EC interactions, at the early stages of tumor vascularization in BC. Indeed, it has been observed that NG2+ early activated pericytes are required not only for pericyte coverage of ECs but also for the deposition of basement membranes that are critical for vessel sprouting formation, maturation, and function [52,53]. Moreover, NG2+ myeloid derived cells, such as tumor-associated macrophages (TAMs) and TIE2-expressing monocytes/macrophages (TEMs), a subset of TAMs close to the luminal surface of blood vessels, participate in early PDGFβ-dependent angiogenesis phases [53]. In the same murine BC model, it has been showed that the depletion of PDGFRβ results in an increased secretion of VEGF, which turns in a diminished pericyte coverage, increased vessel leakiness and metastasis [53]. Thus, it has been suggested that initial steps of the angiogenic process in BC development may be characterized by an increase in PDGFRβ+/NG2+ double positive cells, close to the perivascular niche of the tumor. Concerning the role of PDGFRβ signaling in pericytes and stromal fibroblasts and their relationship with biological parameters and survival, in 512 early BC specimens it has been found that around 35% of tumors are strongly positive for PDGFRβ expression and that this is related with high tumor grade, ER and PR negativity, HER2 expression, proliferation rate, and tumor size [54]. Moreover, high PDGFRβ expression was associated with shorter recurrence-free survival and breast cancer specific survival, mostly in pre-menopausal patients, suggesting a possible role of estrogen in regulating PDGFRβ expression and activity [54]. In an attempt to better differentiate pericyte subtypes and to clear their correlation with tumor vascularization and clinical outcomes, dual pericyte staining for Desmin and PDGFRβ close to CD31+ blood vessels was performed in a small series of TNBC and luminal BC patients (28 and 57 patients, respectively) [55]. TNBC patients showed higher MVD and lower MPI, as compared to luminal subtype, suggesting that in TNBC new vessels are preponderantly immature with reduced pericyte coverage (prevalence of Desmin-/PDGFRβ+ pericytes). At the same time, it has been showed that double-positive pericytes (PDGFRβ+Desmin+) were the dominant pericyte population in Luminal BC patients. These data confirmed previous observations that a higher Desmin+ coverage is associated with vessel stability and reduced lung metastasis [40].

Interestingly, a recent report evaluated PDGFRα, PDGFRβ and PDGF-CC expression of tumor and/or stromal cell in primary tumors (N = 489), synchronous lymph node metastases (N = 135) and asynchronous recurrences (N = 39) using immunohistochemistry in a prospectively maintained cohort of BC patients [56]. Tumor cells displayed high expression of PDGFRα in 20%, and PDGF-CC in 21% of primary tumors, which correlated with the TNBC subtype. Furthermore, patients with high PDGF-CC had a poor prognosis (*p* = 0.04) in terms of 5-year distant recurrence-free intervals, whereas PDGFRα was up-regulated in lymph node metastasis and recurrences compared to primary tumors [56].

### 2.2. Vascular Mimicry

However, angiogenesis is not the only recognized process by which tumors may provide their blood supply. Some tumor cells may generate channels that mimic the function of vessels in a non-angiogenesis dependent pathway (Vasculogenic Mimicry—VM). These tumor cells have a high plasticity that allows them to acquire endothelial cell-like traits. They are periodic acid-Schiff (PAS) positive and endothelial markers (Von Willebrand factor, CD34 or CD31) negative. VM is more commonly associated with late-stage tumors and is frequently activated during cancer metastasis by forming vascular networks. It has been reported that VM is usually present in more aggressive tumor biology and is correlated with poor prognosis [57,58]. A recent meta-analysis, including more than 1200 patients, reported the presence of VM in around 24% of invasive BC cases and a significant correlation with more aggressive tumor phenotype characteristics as well as with poorer prognosis (shorter DFS and OS). No correlations were found with hormone receptor and HER2 status [59]. Conversely, in a murine model of breast tumor, it has been shown that high levels of PAS^+^/CD31^−^ channels significantly correlate with distant metastasis (lung metastasis), but this phenomenon is relevant only for HER2 positive and basal-like/TNBC subtypes [57]. Furthermore, a recent study indicates that HER2-positive BC can exhibit VM in an angiogenic microenvironment after eventually acquiring trastuzumab resistance. Thus, targeting VM might provide a therapeutic benefit to patients with HER2-positive breast cancer [60].

### 2.3. Vessel Co-Option

In addition, several tumors, including BC, develop another alternative tumor blood supply by co-opting pre-existing vessels that can work alone or in association with classical angiogenic ways [61]. Tumor cells, migrating along the preexisting vessels of the host organ, hijack the surrounding normal vessels with preservation of the organ pre-existing architecture. Rather than being a rare event, vessel co-option in humans occurs in around 60–90% of lung metastasis from a variety of tumors [62]. Vessel co-option also represents a significant mechanism of resistance to anti-angiogenic therapy and is prevalent in the majority of BC metastasis to liver, lung, lymph-nodes, skin, and the brain [63,64] confirming the data that primary angiogenic BC can relapse as non-angiogenic tumors [60]. Unfortunately, distinguishing newly formed (angiogenic) vessels from mature “non-angiogenic” vessels, co-opted by the tumor, is challenging by immunohistochemistry or by mRNA profiling [65].

## 3. Metastasis Propensity and Tumor Microenvironment (TME)

**Figure 3:**
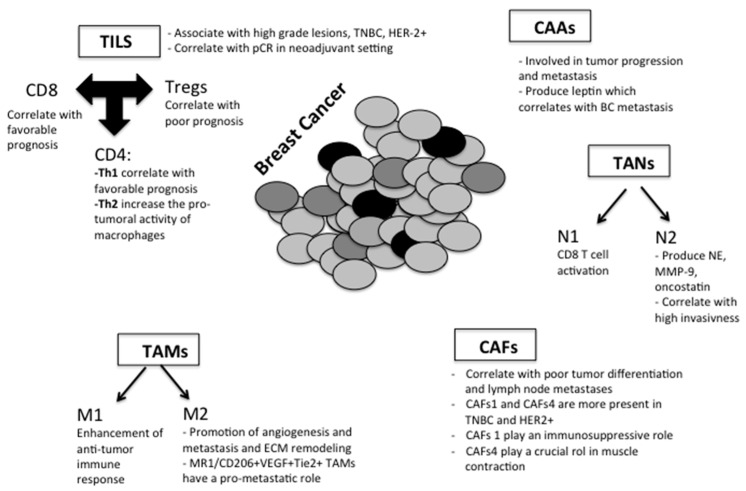
Schematic drawing of the role of cellular and non-cellular components in breast stroma during tumor initiation and progression. Within the tumor microenvironment (TME) there is an array of resident cells, non-resident cells, and secretory elements contributing to the progression and metastasis of breast cancer cells. Tumor-infiltrating lymphocytes (TILs) strongly correlate with good prognosis, in particular in triple-negative and HER2-positive breast cancer patients. T-lymphocytes (CD3+) are the main component of TILs and include CD4+, CD8+ and T-regulatory cells (Treg). Effectively, TILs CD8+ and Th1 cytokines correlate with favorable prognosis, whereas it has been reported a strong association between an increase in Treg-infiltrating and –circulating cells and subsequent breast cancer metastases. Tumor-associated macrophages (TAMs) can express a broad spectrum of activation states. They can have a “classical” tumoricidal phenotype (M1) and an “alternatively” activated tumor-promoting phenotype (M2). It is recognized that perivascular macrophages, involved in the tumor microenvironment of metastasis (TMEM), are a subpopulation of TAMs (Tie2+/VEGF+) with strong pro-angiogenic activity. Similar to macrophages in TME, neutrophils can polarize in two distinct subtypes and N2 tumor-associated neutrophils (TANs) inactivate T-lymphocytes and promote cancer growth. Cancer-associated fibroblasts (CAFs) are the main cell component of breast cancer stroma and are able to secrete a wide range of proteases, inflammatory molecules and growth factors, overall inducing tumor progression and worsening clinical outcome. Cancer-associated adipocytes (CAAs) differ from the normal adipocytes in metabolic activity and leptin production and have been shown to be deeply involved in tumor progression and metastasis.

Within the breast stroma, several “actors” may play crucial roles during tumor initiation and progression and may lead to higher or lower aggressiveness of the disease. Despite the fact that alterations in the breast TME have been recognized as important drivers of tumor progression, the evaluation of several cellular components populating the stroma, but also non-cellular components, such as chemical inflammation mediators, have not been integrated in routine clinical decision making to date (Figure 3). Quantitative and qualitative assessment of different components (cellular and non-cellular) of breast TME at early stages of tumor development may have an important prognostic and predictive value. From this need, many experimental efforts have been performed. A brief description of the many recent findings on the molecular mechanisms underlying the involvement of stromal cells and TME non-cellular elements in early steps of metastatic spread in BC, including epithelial-to-mesenchymal transition (EMT), angiogenesis, extracellular matrix (ECM) remodeling, intravasation and survival in circulation, is presented below (Section 3.1). Then, we focused our attention on the impact of each component of BC stroma on BC aggressiveness, prognosis, and prediction for treatment (Section 3.2).

### 3.1. Molecular Mechanisms of the Influence of TME on Breast Cancer Metastasis

The metastatic process is a complex cascade of events facilitating the spread of cancer cells from the primary tumor site to distal organs. The invasive capability of BC cells is dramatically influenced by the TME, which consists of non-cancer stromal cells, ECM, and soluble factors.

#### 3.1.1. EMT and TME

The detachment of cancer cells from tumor nests and EMT constitute the first steps of the whole metastatic process. The active role played by all stromal cells in inducing EMT is demonstrated by many studies. The mechanisms underlying the destabilization of the epithelial cell–cell junctions, loss of apical-basal polarity, reorganization of the cytoskeletal architecture and an increase capability to invade, as well as to degrade ECM, are correlated with the downregulation of E-cadherin and up-regulation of mesenchymal markers, such as vimentin, N-cadherin, and fibronectin in BC cells [66,67,68]. TGFb, released by tumor-associated macrophages (TAMs) and cancer-associated fibroblasts (CAFs) in the primary TME, has been shown to play a key role in inducing EMT [37,69]. TGFb induces EMT mainly through two pathways: Smads dependent (by activating snail, twist and ZEB transcription factors) and Smads independent pathways (by activating RAS/ERK, JNK and p38 MAPK) [70]. It has been demonstrated that different breast cancer cell lines, grown with conditioned medium, obtained by CAFs isolated from invasive breast cancer tissues, trans-differentiate under TGFb stimulation in a more aggressive phenotype characterized by EMT activation, enhanced cell-extracellular matrix adhesion, migration, and invasion [70]. Besides TGFb, many factors, such as IL-1, TNFa, IL-6 and HIF, released by CAFs and TAMs, are involved in orchestrating the EMT program [67,68]. Furthermore, TAMs are also able to secrete matrix metalloproteinases (MMPs) that can degrade cell-matrix adhesions as well as cell–cell junctions and release EMT regulators by degrading ECM into TME [71]. Recent evidence showed that stromal cells can release microRNAs, such as miR21 and miR200 family members, through exosomes to communicate with cancer cells and induce EMT [72]. Other than CAFs and TAMs, cancer-associated adipocytes (CAAs) may induce EMT in BC cells. Indeed, it has been demonstrated that when human adipose derived stem cells were co-cultured with MCF7 breast cancer cell lines, their paracrine effects activated the EMT program as well as cancer invasiveness [73]. It has recently been revealed that immune cells, recruited by BC cells in TME, may contribute to the EMT process. In a pilot study of 16 breast cancer patients, TNFa, IL-6 and TGFb production by peripheral blood T cells was correlated with the detection of circulating tumor cells expressing EMT markers [74].

#### 3.1.2. Angiogenesis and TME

Stromal cells recruited in breast TME play a crucial role in promoting angiogenesis, in concert with BC cells. CAFs are the main source of many pro-angiogenic factors, including VEGF, PDGFC and osteopontin [75]. A recent study showed that hypoxia inducing the expression of HIFa in CAFs caused release of VEGFA and promoted the formation of tubule-like structures in HUVEC cell line [76]. A great contribution in angiogenesis process is also made by TAMs [77]. Depletion of TAMs resulted in the inhibition of tumor angiogenesis, whereas reconstitution of TAMs promoted angiogenesis in a murine breast cancer model [78]. A brilliant study revealed that the pro-angiogenic factor angiopoietin 2 (ANG2) not only stimulated endothelial cells but also macrophages expressing its receptor TIE2 to actively participate in the formation of vessel networks in BC [79]. Moreover, it is well established that ECM secreted by CAFs, usually, results in a hypoxic environment inside the tumor, inducing the recruitment and activation of TAMs with increased production of HIFa and pro-angiogenesis factors, which ultimately lead to neo-angiogenesis [80]. Hypoxic conditions in the TME contribute to Treg proliferation by CCL28 and VEGF overexpression in tumor cells. Tregs secrete VEGF, recruit endothelial cells, and promote tumor angiogenesis directly. Furthermore, Tregs indirectly facilitate tumor angiogenesis by inhibiting Th1 cell activation and polarizing TAMs into the M2-like phenotype [81,82]. Recently, it has been demonstrated that Th17, a subtype of CD4 positive T helper cells, promote endothelial cell proliferation and tumor angiogenesis by expressing IL-17, a poor prognostic factor in BC [83,84]. Among immune cells, neutrophils are known to play an important role during neo-angiogenesis in tumors. Conclusive evidence of neutrophil involvement in tumor angiogenesis came from studies in the RIP-Tag2 multi-step pancreatic carcinogenesis mouse model: indeed, their depletion using anti-GR1 antibodies reduced the number of dysplastic islets that were undergoing angiogenesis [85]. It has also been reported that STAT3 activation in neutrophils triggers the angiogenic switch through secretion of multiple angiogenic factors, such as VEGF, IL-8, TNFa, MMP9, bFGF and Ang1 in mice [86], and that human neutrophils uniquely release TIMP (tissue-inhibitors of metalloproteinases)-free MMP-9 to provide an important stimulation of angiogenesis [87].

#### 3.1.3. ECM Remodeling and TME

ECM contributes to actively support metastatic spread of breast cancer through the crosstalk between stromal and tumor cells. CAFs are the key regulators involved in the aberrant ECM remodeling: they secrete collagen, tenascin C and fibronectin and, at the same time, produce metalloproteinases (MMPs) that lead to ECM degradation [88]. During transformation of ductal carcinoma in situ to invasive ductal cancer, there is an enhancement of the collagen fibers cross-linking, as well as their thickening and linearization, driving to ECM stiffening, that, in turn, activates pathways regulating focal adhesion, TGFb signaling and pro-tumorigenic macrophage infiltration [89,90]. In addition, it has been demonstrated that the tumor hypoxic microenvironment led to a high expression of lysyl oxidase (LOX) in CAFs with enhanced cross-linking enzymatic activity, creating the molecular tracks that pave the way for BC cells, to migrate beyond the primary tumor [91]. CAFs are able to produce the multimeric extracellular glycoprotein tenascin-C, which correlates with tumor stage, lymph node metastasis, TAM infiltration and predicts poor survival [92]. As mentioned before, CAFs role in remodeling ECM is connected with their production of several MMPs. Indeed, it has been shown that an enhanced BC cell migration is promoted by higher production of stromal MMP-9 via the release of TGFB and TNFa [93]. Recent studies observed that TAMs actively cooperate with CAFs; they secrete the protein, acidic and rich, on cysteine (SPARC), involved in regulating cell-ECM interactions, and move along with BC cells on fibrillar collagen 1 structures toward blood vessels [94].

#### 3.1.4. Intravasation/Survival in Circulation and TME

Intravasation is the process by which tumor cells migrate into blood or lymph vessels through trans-endothelial migration. There are two kinds of intravasation: transcellular (tumor cells migrate across the body of the endothelial cells that undergo degradation of skeleton and contraction) and paracellular (tumor cells migrate across the vessel walls by opening the junctions between endothelial cells) [95,96]. In transcellular intravasation CAFs exert an active role, producing CXCL12 and TGFb that induce the proliferation of tumor cells and protect them in the tumor embolus, a nest of tumor cells embedded in endothelium [97]. Furthermore, it has been showed that CAFs secrete CXCL12 that act on endothelial cells through receptor CXCR4 and decrease the expression of tight junction molecules, promoting paracellular intravasation [98]. However, in the paracellular intravasation, TAMs usually play a key role interacting with tumor cells and endothelial cells, creating a peculiar cellular microstructure called tumor microenvironment of metastasis (TMEM), that is well described in Section 3.2.1. [17]. Following the intravasation, BC cells are referred to as circulating tumor cells (CTCs) and in vitro and in vivo studies have shown that CAFs can induce the formation of tumor cell clusters, secreting stromal cell-derived factor 1 (SDF1) and TGFb, via Src activation [98]. The potential clinical role of breast CAFs in cancer cell cluster formation and metastasis progression is also supported by findings indicating the presence of BC inflammatory emboli characterized by increased cell—cell adhesion with high E-cadherin expression and the hybrid epithelial/mesenchymal state [99]. A recent study speculated that the gene expression profile of CAFs changes with the metastatic disease, suggesting that the transcriptional evolution of these cells is fundamental for the generation of a metastatic niche [100]. Recently, it has also been suggested that neutrophils, via neutrophil-derived leukotrienes, aid the colonization of distant tissues though selective expansion of the sub-population of CTCs that retain higher metastatic potential [101]. In addition, a very interesting study showed that CTCs with an epithelial-to-mesenchymal transition phenotype positivity and elevated neutrophil-to-lymphocyte ratio (NLR) were associated with shorter PFS in 284 primary BC patients [102].

### 3.2. Tumor-Microenvironment Components

#### 3.2.1. Tumor-Associated Macrophages (TAMs)

Unlike tissue-resident macrophages, which originate from the yolk sac, in BC macrophages derive largely from circulating monocytes. Their normal function is to promote both innate and adaptive immunity, but BC largely re-educates them to phenotypes that promote tumor growth and spread. Indeed, they may acquire different specialized activities, depending on the distinct signals they are exposed to in different tumor microenvironments, such as perivascular, stromal, or necrotic tumor areas [23]. Consequently, TAMs express a broad spectrum of activation states between the two extreme forms of “classical” tumoricidal phenotype (M1) and “alternatively” activated tumor-promoting phenotype (M2), with a tendency toward the latter [23]. More than 15 years ago, using intravital multiphoton imaging in rat and mouse mammary tumors, it was found that “specific” invasive tumor cells, after polarizing, move toward and invade blood vessels only in association with perivascular macrophages [103]. Subsequently, on the basis of this observation, an interesting study implicating the use of a mouse mammary cancer model found that tumor cells overexpressing Mena, a cytoskeletal regulatory protein, in conjunction with a perivascular macrophage and an endothelial cell, constituted a microanatomic landmark of a portal leading to carcinoma cell intravasation (termed tumor microenvironment of metastasis—TMEM). This microstructure has been found to constitute the restricted area where a transient loss of vascular junctions determines a focal and temporal limited vascular permeability site, allowing tumor cells intravasation. Thus, vascular permeability to tumor cells has been suggested as dynamic, localized, and restricted to the TMEM [104]. Interestingly, TMEM density showed a positive correlation with high tumor grade and distant metastasis propensity [105]. Afterwards, in a subsequent retrospective study performed on 600 patients with stage I-III breast cancer treated with adjuvant chemotherapy in trial E2197 (NCT00003519), plus endocrine therapy for HR+ disease, TMEMs were enumerated using an analytically validated, fully automated digital pathology/image analysis method (MetaSite *Breast*^TM^). TMEM score was positively associated with distant recurrence-free interval (*p* = 0.001) and recurrence-free interval (*p* = 0.00006) in ER+/HER2- BC patients within the first 5 years of follow-up after surgery, suggesting a possible role in providing prognostic information for early recurrence complementary to clinicopathological features and recurrence score [17,106]. In the same study, this correlation was not observed in TNBC and HER2+ BC subtypes, and TMEM structures in BC can be identified and quantified in formalin-fixed paraffin-embedded tissue by using triple immunostaining for the three different cell types recognized in direct physical contact, and precisely, Mena-overexpressing tumor cells (pan-Mena antibody), macrophages (CD68), and endothelial cells (CD31). It is recognized that perivascular macrophages involved in TMEM are a subpopulation of TAMs (Tie2+/VEGF+) with strong pro-angiogenic activity. Indeed, they secrete large amounts of VEGF-A and induce a transient blood vessel permeability [107], attracting tumor cells, the only ones possessing adequate motility derived from the high expression of Mena isoforms, and facilitating their dissemination into the blood stream [104]. More recently, in a mouse model of HER2+ BC, it has been shown that TMEM containing TAMs (Tie2+/VEGF+) attracted from the stroma into the ductal epithelium are also present in pre-malignant lesions and favor dissemination of tumor cells much earlier than in local tumor growth [108].

Moreover, several clinical studies have shown that high macrophage numbers in primary BC are associated with a worse outcome, indicating, although indirectly, that TAMs, mainly those with high expression of MRC1/CD206 (the mannose receptor C-type lectin) and positive for VEGF and Tie2, may have a pro-metastatic role [109]. Interestingly, a recent study demonstrated that the infiltration of TAMs overexpressing cytochrome P450 (CYP) 4A was positively associated with pre-metastatic niche formation, metastasis, as well as poor prognosis in breast cancer patients [110].

A subset of innate immune cells, called myeloid-derived suppressor cells (MDSCs), also contribute to BC metastasis. It has been demonstrated that MDSCs, attracted in primary tumor site by BC cells through CCL3 chemokine, produce IL-10, TGFb and VEGF, suppressing immune response, increasing epithelial-to-mesenchymal transition (EMT), and stimulating angiogenesis, respectively [111].

#### 3.2.2. Tumor-Infiltrating Lymphocytes (TILs)

Of note, we now have evidence that TILs are clinically meaningful, as their quantification in the intratumoral stroma strongly correlates with good prognosis, in particular in triple-negative and HER2-positive breast cancer patients. TILs constitute a continuous variable quantified as a percentage of area occupied by TILs/total stroma area on hematoxylin- and eosin-stained tumor sections [112]. A training website has also been developed in order to correctly report TILs (www.tilsinbreastcancer.org 4 October 2021). Lymphocyte-predominant BC cases, which have >50% of stromal area infiltrated by TILs, account for about 11% of all invasive breast carcinomas [113] Several studies did show that TILs are more frequently observed in high-grade lesions, in TNBC and HER-2 positive BC [114,115]. Furthermore, it has also been demonstrated that high levels of TILs correlate with pathological complete response (pCR) rates in the neoadjuvant setting in triple-negative and HER-2 positive cancers [116,117]. Although the extent of data on TILs in BC are quite robust, their biological role in different subtypes and their prognostic and predictive value need to be further clarified. In diagnostic practice, TILs are assessed on a mere count on morphological grounds with a not yet brilliant reproducibility, but we are aware that they represent a heterogeneous aggregate of immune cells with different phenotypes, and different roles in inflammatory response. T-lymphocytes (CD3+) are the main component of TILs and include CD4+, CD8+ and T-regulatory cells (Treg). Effectively, TILs CD8+ and Th1 cytokines correlate with favorable prognosis, whereas it has been reported a strong association between an increase in Treg-infiltrating and –circulating cells and subsequent breast cancer metastases [23,118,119].

#### 3.2.3. Neutrophils

Neutrophils represent 50–70% of the leukocytes present in human blood and are the main cells of innate immunity. Similar to macrophages, in TME, neutrophils can polarize in two distinct subtypes and N2 tumor-associated neutrophils (TANs) inactivate T-lymphocytes and promote cancer growth [23,120]. TANs are supposed to convert disseminated dormant tumor cells in metastatic cells through the release of neutrophil elastase (NE), matrix metalloproteinase-9 (MMP-9), and oncostatin-M, leading to tumor cell proliferation and invasiveness [23,120]. The recruitment of neutrophils in TME is mainly regulated by chemokine receptor CXCR2 and it has been reported that TNFa-activated mesenchymal stem cells (MSCs) releasing CXCL1, CXCL2 and CXCL5 efficiently recruited CXCR2 positive neutrophils into the tumor, promoting BC metastasis [121]. A very brilliant study showed that circulating tumor cells (CTCs) could associate with neutrophils in breast cancer and that the formation of these clusters causes an enhancement of metastatic potential of CTCs [101]. Interestingly, recent evidence demonstrates that an increased neutrophil-to-lymphocyte ratio (NLR) assessed in blood samples, may have a negative predictive and prognostic role in the early setting of all BC subtypes [122]. A recent retrospective study investigated the role of NLR in BC patients receiving neoadjuvant chemotherapy and showed that the pCR rate in patients with low pre-treatment NLR (NLR < 2.06) was higher than in those with higher NLR (NLR > 2.06) (25.5% vs. 14.3%; *p* < 0.05). In addition, high NLRs were an independent significant predictor of lower breast cancer-specific survival in patients undergoing neoadjuvant chemotherapy [122].

#### 3.2.4. Cancer-Associated Fibroblasts (CAFs)

CAFs are the main cell component of BC stromal compartment and are able to secrete a wide range of proteases, inflammatory molecules, and growth factors, inducing tumor progression and metastasis by promoting cancer cell growth, pro-tumor immune responses, extracellular matrix (ECM) remodeling and angiogenesis, and worsening clinical outcome [123]. CAFs may originate from resident fibroblast but also from other cell types, such as mesenchymal stem cells, cancer cells, cancer stem cells and endothelial cells, through a process known as trans-differentiation [124]. Recently, an interesting study demonstrated that potent pro-inflammatory cytokines, such as tumor necrosis factor (TNFα) and interleukin-1β (IL-1β), that are present in the TME of breast primary tumors, may lead to the conversion of mesenchymal stem cells to tumor-promoting inflammatory CAFs. These inflammatory CAFs release factors, such as TGFβ-1, C-X-C motif chemokine ligand 12 (CXCL12), PDGF and IL-6, that enhanced tumor cell dispersion, scattering, and migration, by stimulating the chemokine receptors CCR2, CCR5, and CXCR1/2 and Ras-activating receptors, expressed by BC cells [125]. At the same time, these cytokines also induce the formation of CAFs from other cell types in the TME via trans-differentiation. BC cells are also able to drive the differentiation of mesenchymal stem cells to fibroblasts through the production of osteopontin [126]. In 2018, a meta-analysis, including 3680 breast cancer patients from 15 published studies, found that a high density of tumor-infiltrating fibroblasts was associated with poor tumor differentiation and lymph node metastasis [127]. Moreover, it has been suggested that CAFs may promote a mesenchymal phenotype acquisition both in pre-malignant and malignant mammary cells through deposition of a distinct extracellular matrix (ECM), characterized by aligned collagen fibers/deposit [23]. The presence of this specific ECM, observed by scanning electron microscope, may allow us to identify primary BC with high propensity to early metastatic spread. CAFs subsets were recently identified by multicolor flow cytometry: CAFs1 are involved in ECM organization and maintain an immunosuppressive microenvironment, especially in HER-2 positive carcinomas and TNBCs [128]; CAFs2 are equally distributed in intra-tumoral and extra-tumoral stroma and are enriched in Luminal A carcinomas; CAFs4 are more present in HER-2 positive carcinomas and TNBCs and play a crucial role in muscle contraction [128].

#### 3.2.5. Cancer-Associated Adipocytes (CAAs)

CAAs are activated in TME by BC cells and show different features from adipocytes present in normal adipose tissues. They are smaller for their reduced lipid content and are able to produce high levels of pro-inflammatory cytokines, in particular IL-6 and leptin, that have been shown to promote the growth, invasion and metastasis of BC cells [129,130]. Two recent studies demonstrated the role of CAAs during BC metastasis. One showed that plasminogen activator inhibitor-1 (PAI-1), released by BC cells, is required to activate lysyl hydrolase-2 (PLOD-2) in CAAs, resulting in collagen remodeling and metastasis formation [131]; the other one reported that obesity causes an increase in TAMs that secreting IL-1b activate adipocytes to produce angiopoietin-like 4 (ANGTL4) that in turn promotes angiogenesis in breast cancer [132]. In addition, CAAs have been shown to provide high-energy metabolites, such as pyruvate, lactate, ketone bodies, and fatty acids, to BC cells in order to use them as energy source [133].

#### 3.2.6. Cytokines

Several cytokines are involved in BC angiogenesis, immunosuppression and promotion of metastasis and have been correlated with tumor stage, survival, and malignancy, rendering them potential prognostic factors [134] (Table 2).

TGFβ is the most extensively studied cytokine in BC. It enhances tumor vascularity by regulating the expression of VEGF, promotes immune evasion and ECM degradation, and its high plasma levels might be predictive of early relapse of invasive BC and lymph node metastasis [135,136]. It has also been documented that stromal fibroblasts and CAFs are an important source of TGFβ, leading to the initiation of the epithelial-to-mesenchymal transition (EMT) process, which finally ends with the spreading of BC [37].

At the same time, an enormous amount of data showed an association between high serum levels of IL-1β ((especially in ER negative cancers), IL-6, TNFα, IL-8 (particularly in HER2 positive cancers), invasiveness, and poor prognosis [134,137,138,139,140,141,142].

Leptin, a pro-inflammatory cytokine, is secreted primarily by adipocytes and its expression correlates with the stage of invasion. Cancer-associated adipocytes (CAAs) differ from the normal adipocytes in metabolic activity and adipokines expression and have been showed to be deeply involved in tumor progression and metastasis. An overview of systematic reviews explored the association between circulating leptin levels and risk of BC. Higher levels of leptin were found to be associated with an increased risk of premenopausal and postmenopausal BC, suggesting a possible use of this biomarker in a comprehensive risk predictive score for female BC [143,144]. Moreover, leptin receptor (ObR) expression was found to be associated with ER expression and tumor size, suggesting an interaction between the leptin and estrogen systems to promote breast carcinogenesis [145]. However, there are currently no definitive data on this topic and further prospective and large studies need to be conducted.

## 4. Conclusions

In BC, the composition and the architectural organization of the mammary gland parenchyma is subverted. In this context, the TME exerts a central role in cancer onset and spread. TME has been richly studied in BC and it is now recognized as an important element that deeply affects cancer biological behavior, response to treatment and patient prognosis.

Although BC is among the most investigated tumors, few comparable data are available regarding the association between tumor vasculature and microenvironment phenotype at the early stages, as well as clinical outcomes in order to integrate the prognostic and predictive value derived from the assessment of hormone receptors (ER and PR) and of HER2 status.

Future methodologically correct studies are needed in BC, in order to incorporate TME features in diagnostic algorithms.

## Figures and Tables

**Table 1:** Correlation between MVD, pMVD and VPI and prognosis in breast cancer Luminal A and basal-like subtypes.

	Mean Differences/ (95% CI)	*p*	Hazard Ratio (95% CI)	*p*
MVD, microvessels/mm^3^ Luminal A Basal-like	7.5 (−6.1–21.2)	>0.05		
pMVD, microvessels/mm^3^ Luminal A Basal-like	1.9 (0.7– 3.1)	0.002 *		
VPI, percentage points Luminal A Basal-like	1.7 (0.3–3)	0.014 *		
MVD, microvessels/mm^3^ Luminal A per 10 vessels increase Basal-like per 10 vessels increase			1.22 (1.09–1.37) 1.04 (0.95–11.5)	<0.001 * >0.05
pMVD, microvessels/mm^3^ Luminal A per 1 vessel increase Basal-like per 1 vessel increase			1.11 (0.86–1.43) 1.04 (0.96–1.14)	>0.05 >0.05
VPI, percentage points Luminal A per % point increase Basal-like per % point increase			0.98 (0.83–1.16) 1.02 (0.91–1.13)	>0.05 >0.05

MVD: Microvascular density; pMVD: proliferating microvascular density; VPI: vascular proliferation index. * Statistical significance

**Table 2:** Cytokine levels and breast cancer development.

Cytokine	Levels	Environment	Function	Impact on Prognosis
TGFβ	++	tumor/serum	enhances tumor vascularity, promotes immune evasion and ECM degradation	early relapse and metastases worse survival
IL-1β	++	tumor/serum	enhances tumor vascularity, inhibits apoptosis in cancer cells, downregulates ER	not determined
IL-6	++	serum	promotes EMT and tumor aggressiveness (inhibits response to chemotherapy)	worse survival
TNFα	++	serum	inhibits apoptosis in cancer cells	not determined
IL-8	++	serum	enhances endothelial cell proliferation and MMP production	not determined
Leptin	++	serum	promotes breast carcinogenesis	not determined
IL-10	++	serum	promotes immune evasion	not determined

Transforming growth factor β: TGFβ; Interleukin 1β: IL1β; Interleukin 6: IL6; tumor necrosis factor α: TNFα; Interleukin 8: IL8; Interleukin 10: IL10. ++: overexpression as compared to control.
